# Shape Transformations of Lipid Vesicles by Insertion of Bulky-Head Lipids

**DOI:** 10.1371/journal.pone.0132963

**Published:** 2015-07-15

**Authors:** Soichiro Tsuda, Tatsuya Sakakura, Satoshi Fujii, Hiroaki Suzuki, Tetsuya Yomo

**Affiliations:** 1 Yomo Dynamical Micro-scale Reaction Environment Project, ERATO, Japan Science and Technology Agency, 1-5 Yamadaoka, Suita, Osaka, 565-0871, Japan; 2 Graduate School of Information Science and Technology, Osaka University, 1-5 Yamadaoka, Suita, Osaka, 565-0871, Japan; 3 Graduate School of Frontier Biosciences, Osaka University, 1-5 Yamadaoka, Suita, Osaka, 565-0871, Japan; Universidad del Pais Vasco, SPAIN

## Abstract

Lipid vesicles, in particular Giant Unilamellar Vesicles (GUVs), have been increasingly important as compartments of artificial cells to reconstruct living cell-like systems in a bottom-up fashion. Here, we report shape transformations of lipid vesicles induced by polyethylene glycol-lipid conjugate (PEG lipids). Statistical analysis of deformed vesicle shapes revealed that shapes vesicles tend to deform into depended on the concentration of the PEG lipids. When compared with theoretically simulated vesicle shapes, those shapes were found to be more energetically favorable, with lower membrane bending energies than other shapes. This result suggests that the vesicle shape transformations can be controlled by externally added membrane molecules, which can serve as a potential method to control the replications of artificial cells.

## Introduction

Lipid vesicles, in particular Giant Unilamellar Vesicles (GUVs) have long been employed as model cell membranes to elucidate the physicochemical basis of biological cellular functions. While even simplest bacterial cells are still complex, lipid vesicles are simple enough and amenable to alteration of membrane compositions and inner aqueous contents. They have been used in biophysical studies investigating, for example, lipid raft formation, [1, 2] interaction with small molecules and peptides, [3, 4] and morphological transformations upon external stimulations. [5, 6] In recent years, lipid vesicles are increasingly used as compartments of artificial cells, or “protocells”, to reconstruct living cell-like systems in a bottom-up fashion. So far, protein expression, [7, 8] cascade reaction, [9] RNA self-replication, [10] directed evolution of an enzyme [11] and membrane protein, [12, 13] programmed vesicle fusion [14, 15] and reconstruction of membrane protein complex [16] have been demonstrated.

In parallel with those studies on the internal content of artificial cells (i.e., replication of information molecules and metabolism), several studies have been carried out to achieve growth and division of artificial cells (i.e., replication of compartments). Andes-Koback and Keating demonstrated that lipid vesicles encapsulating Aqueous Two-Phase System (ATPS) exhibited asymmetric budding and division induced by dehydration. [17] Tanaka et al. showed that addition of lysophosphatidylcholine (lysoPC) micelles induced budding and fission of lipid vesicles. [18] Recently, we reported fusion and division cycles of lipid vesicles containing polyethylene glycol (PEG) molecules using electrofusion and spontaneous budding. [19, 20] It was also shown that such shape transformation could occur with non-lipid vesicles, such as fatty acid vesicles [21] and synthetic amphiphile vesicles. [22]

What is still missing for the growth and division of artificial cells is a method to robustly control morphological transformations of lipid vesicles. Due to the extreme sensitivity to bilayer asymmetry [23] and low bending moduli of lipid membranes, [24] various types of shape transformations can be observed upon a single external stimulus, which makes it difficult to assess the effect of the external stimulus on vesicle shapes. To address this issue, we here take a statistical analysis approach for vesicle shape transformations to quantitatively understand the effect of external stimulus. [25] In particular, we use phospholipids conjugated with PEG molecules and show that cell division-like budding transformations of lipid vesicles can be triggered by insertion of the PEG lipids into vesicle membranes. PEG lipids are used as an extreme case of bulky head lipids, with which flip-flop from the outer to the inner membrane does not occur and will stay on the outer membrane once incorporated. PEG lipids are commonly used to stabilize drug delivery liposomes [26] and chemical properties are well studied. [27, 28]

In contrast to previous studies on the shape deformation of vesicles, which mostly focused on tracking temporal shape changes of individual vesicles, we statistically analyzed a number of vesicle shapes sampled from microscope snapshot images. An advantage of this statistical analysis over conventional methods is that the analysis gives us average and variational information on vesicle transformation dynamics. For instance, the statistical analysis revealed that there were meta-stable shapes in osmotically-deformed lipid vesicles. This would be difficult to find out with conventional approaches of observing single instances of temporal shape transformations.

Here, the ratio of long and short axes of lipid vesicles is used as a measure of shape deformation. We employed this measure because theoretically it is known that the shape of vesicle becomes elongated as the area difference between inner and outer leaflet increases [29] (see also S1 Fig for example). Thus, we hypothesized that vesicles should deform into more elongated shapes from spherical ones if PEG lipids are incorporated into vesicles and the area of outer layer of vesicle membrane is increased. The average and histograms were plotted to study the statistical behavior of morphological changes. Although shape transformations of vesicles induced by external stimuli have been reported previously, only a few studies reported statistical analysis of lipid vesicles. [25, 30, 31] This is partly because of difficulties in preparing an initial population of vesicles in a similar condition (e.g. lamellarity, shape) using conventional preparation methods. We have overcome this issue with a new vesicle formation method using water-in-oil (W/O) emulsions. [32, 33] Lipid vesicles formed by this method were reported to be mostly unilamellar, [34] and the shapes were mostly spherical. [25]

## Methods

### Materials

1-palmitoyl-2-oleoyl-sn-glycero-3-phosphocholine (POPC) and 1-palmitoyl-2-oleoyl-sn-glycero-3-[phospho-rac-(1-glycerol)] (POPG), 1,2-distearoyl-sn-glycero-3-phosphoethanolamine-N-[poly(ethylene glycol)2000-N’-carboxyfluorescein] (DSPE-PEG(2000) CF), 1,2-distearoyl-sn-glycero-3-phosphoethanolamine-N-[biotinyl(polyethylene glycol)-2000] (DSPE-PEG(2000) Biotin. Hereafter referred to as “PEG lipid” for short) were purchased from Avanti Polar Lipids (Alabaster, USA). Cholesterol was purchased from Nacalai Tesque (Kyoto, Japan). These lipids were stored in a freezer at -20°C to avoid any degradation of lipids and dissolved in chloroform (100mg/mL concentration) prior to experiments. Fluorescent proteins, transferrin from human serum Alexa Fluor 647 conjugate (TA647, for short) and Alexa Fluor 488 Fluorescent Streptavidin Conjugates (Avidin AF488) were purchased from Invitrogen (Carlsbad, USA). Aqueous solution encapsulated in giant vesicles contains 50 mM HEPES-KOH (pH 7.6), 200 mM sucrose, and 1.5 *μ*M TA647. Buffer solution contains 50 mM HEPES-KOH (pH 7.6), 200 mM glucose.

### Preparation of Giant Lipid Vesicles and PEG Lipid Suspensions

Lipid vesicles are prepared by the W/O emulsion transfer method [34]: First, POPC, POPG, and cholesterol in chloroform was mixed at 9:1:0.5 weight ratio and dissolved in liquid paraffin. Chloroform was then evaporated in the oven at 80°C for at least 20 min. The aqueous solution to be encapsulated in lipid vesicles and lipid/paraffin solution were mixed at 1:10 volume ratio and thoroughly vortexed for 30 s. The mixture was settled on ice for 10 min to stabilize the W/O emulsions. It was then gently placed on top of the buffer solution and centrifuged at 4°C, 18,000 x g for 30 min. Vesicles were formed when W/O emulsions pass through the interface between the paraffin mixture and the buffer solution. For further details, see elsewhere. [34] A buffer solution containing PEG lipid micelles was prepared by following protocol: First, 1 mg of PEG lipids dissolved in 10 *μ*L of chloroform were added to 1 mL of the buffer solution and vortexed for 30 s. The mixture was set in an ultrasonic bath and sonicated for 30 min. After lightly agitated to disperse small chloroform emulsions, it was then kept in an oven at 80°C for 30 min to evaporate chloroform and to form PEG lipid micelles. Pure Milli-Q water was added to make up for water lost during the evaporation step (Final concentration 331 *μ*M).

### Microscope Observation and Image Analysis

Lipid vesicles were observed using a differential interference contrast (DIC) microscope (Eclipse Ti-U, Nikon, Japan) and laser scanning confocal microscope (IX71, Olympus, Japan, equipped with a confocal scanning unit, CSU-X1, Yokogawa Electric). The former microscope was used to observe temporal shape transformations of lipid vesicles due to the lipid insertion. The latter was used to obtain fluorescent snapshot images of vesicles, which will be used for shape analyses of transformed lipid vesicles. Prior to confocal microscope observations, a buffer solution containing PEG lipid micelles was mixed with a solution containing lipid vesicles at 1:1 volume ratio. The final concentrations of the micelles (hereafter, PEG lipid concentration) were 165, 82.5, 41.25, 20.63, 10.32, 5.16, 2.58 *μ*M, and 0 *μ*M (the buffer solution only, as control experiment). In all conditions, the PEG lipid is forming micelles because the critical micelle concentrations (CMC) of DSPE-PEG(2000) in buffer solution was known to be around 0.5–1 *μ*M. [26] The mixture was left to settle for 30 min on a glass slide and observed under microscope for 5min, unless otherwise stated. As lipid vesicles encapsulate 200 mM sucrose while the outer buffer solution contain 200 mM glucose, lipid vesicles sank down to the bottom of the glass slide. Thus, the major axis of vesicles were expected to be in parallel with the horizontal plane of the microscope. Multiple images with different Z-height were taken at each XY position.

**Fig 1:**
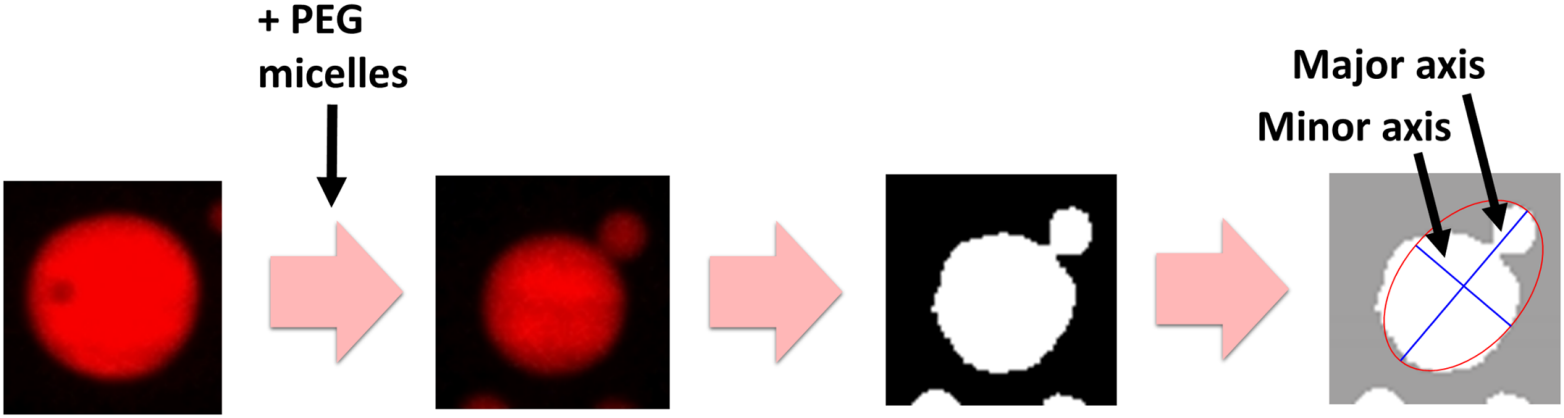
A schematic for vesicle image analysis. Vesicles are reacted with PEG lipid micelles to induce shape transformations and images are taken by a confocal microscope. Each vesicle image was binarized separately and approximated with an ellipsoid to measure the lengths of major and minor axes.

Obtained confocal images of lipid vesicles were analyzed using a custom software on Matlab. Fig 1 shows a schematic for the image analysis. Vesicles of 5 to 20 *μ*m in diameter were used for analysis and other vesicles were omitted. To analyze the shape of a lipid vesicle, a Z plane that maximizes the size of the vesicle was manually selected and binarized using adaptive histogram equalization algorithm. Vesicles were approximated as ellipsoids and the lengths of major and minor axes were measured. In total, more than 300 vesicles were analyzed for each PEG lipid concentration.

Three dimensional model of vesicle shapes was reconstructed from a stack of microscope images to calculate the reduced volume of temporally-deforming lipid vesicles. After addition of PEG lipid micelles to the solution containing lipid vesicles, lipid vesicles are scanned along Z-axis by taking images with 1 Âμm gap using motorized stage. For 3D reconstruction, a stack of single lipid vesicle images was first binarized and the surface of the vesicle is reconstructed based on the black-and-white images using surface triangulation on Matlab.

### Theoretical calculations of vesicle shapes

Vesicle shapes at different membrane and internal volume conditions were simulated using the Bilayer-Coupling (BC) model. [29] We chose the BC model among other theoretical models for vesicle shapes because the observed shape transformations of lipid vesicles appeared to be continuous (i.e., second-order transition) as they gradually transform from spherical to budded shapes via prolate shape. The BC model is suitable for modeling such transformation, in contrast to discontinuous transformations, for which the spontaneous curvature (SC) or area-difference elasticity (ADE) models are more suitable. To numerically calculate vesicle shapes, we used the Spherical Harmonics Parameterization (SHP) method. [25, 35] A bending energy function for the BC model is:

$$W_{b}/8\pi \kappa_{b}=w_{b}=\frac{1}{2}\oint {\left(C_{1}+C_{2}\right)}^{2}dA$$

where *W*_*b*_ is the membrane bending energy, *w*_*b*_ the bending energy relative to a sphere, *κ*_*b*_ the bending rigidity, *C*_1_,*C*_2_ local mean curvatures, and A is the total surface area. The energy function is minimized under two constraints: (1) area difference Δ*a* = *M*/4*πR_A_* ^2^, (a difference between the areas of inner and outer membrane leaflets), where *M* = 1/2 ∮(*C*_1_+*C*_2_)*dA* and $R_{A}=\sqrt{A/4\pi }$, and (2) reduced volume *v* = *V*/(4*π*/3)*R_A_* ^3^ (the volume of a vesicle relative to a sphere). The minimization process was carried out on Matlab as a parameter optimization using active set algorithm. Further details can be found elsewhere. [25, 35]

## Results and Discussion

### Shape Transformations of Giant Vesicles

**Fig 2:**
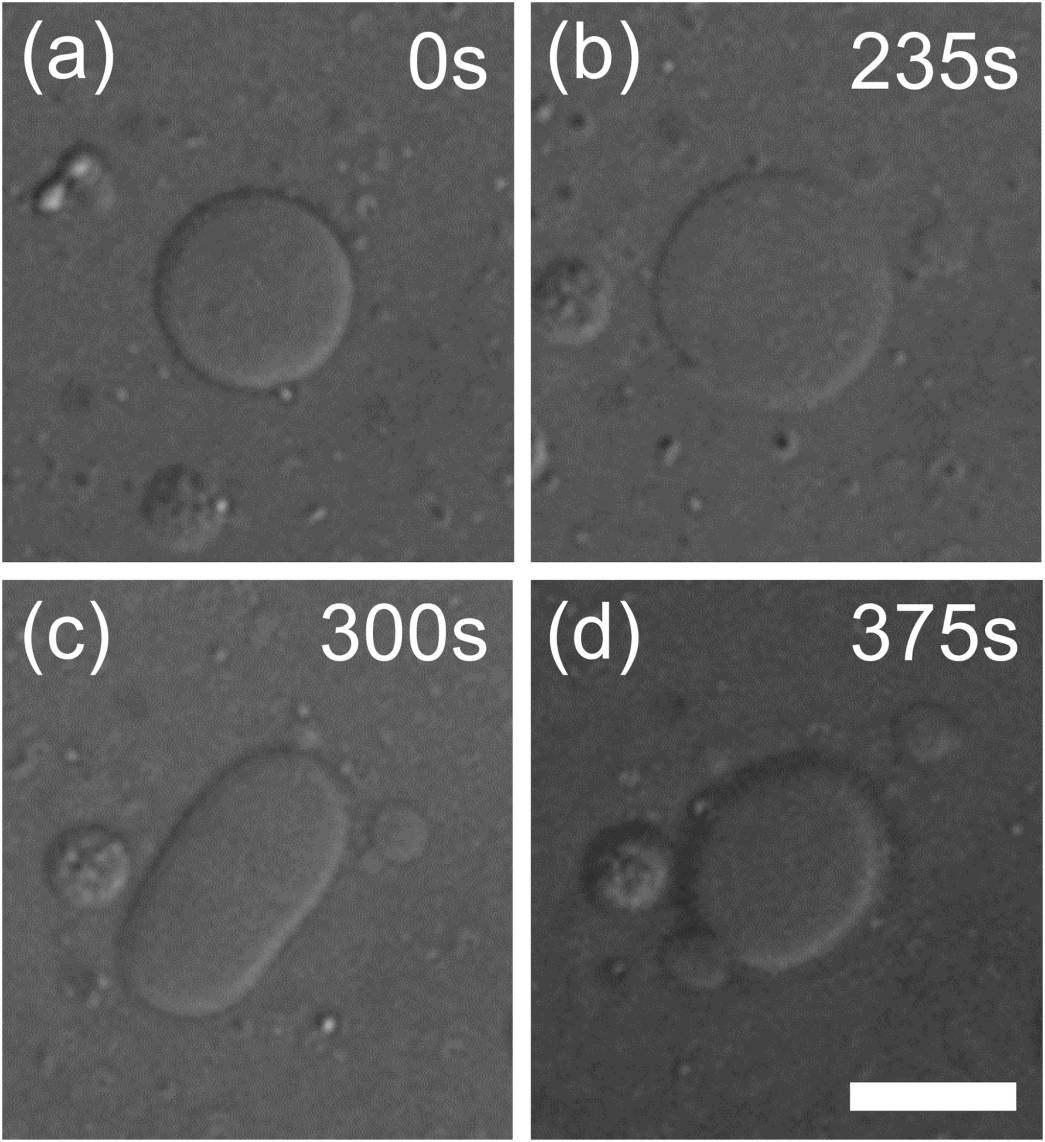
A shape transformation of lipid vesicle induced by PEG lipid micelles. (a) A spherical vesicle transformed into (b) a disc-like oblate, (c) cylindrical prolate, and eventually divided into (d) two large and small vesicles.

Fig 2 shows a typical shape transformation of a lipid vesicle induced by fusion of PEG lipid micelles (see also S1 Video). Before addition of the micelles, most of the lipid vesicles were, in general, circular (e.g. Fig 2a). Upon micelle addition, vesicle diameter increased, which suggests that the vesicles were transformed into oblates (i.e., disc-like shape, Fig 2b). As this transformation progressed, the vesicles turned into prolates (i.e., cylindrical shape, Fig 2c) and finally divided into two daughter vesicles (Fig 2d). Most, if not all, vesicles showed similar transformations regardless of the micelle concentrations. This series of shape transformations were observed in both cases when fluorescent PEG lipid micelles (DSPE-PEG(2000) CF) and biotinylated PEG lipid micelles (DSPE-PEG(2000) Biotin) were added. However, most of shape transformations stalled either in oblate or prolate shapes in the case of fluorescent PEG lipids, even at the highest micelle concentration. Vesicles divided into two vesicles were observed only occasionally. In contrast, in the case of biotinylated PEG lipids, a larger portion of vesicles exhibited division into two or more daughter vesicles at the higher micelle concentrations tested and the transformations appeared to have completed within 15 min after the addition of PEG lipid.

We speculated that the different results with the fluorescent and biotinylated PEG lipids were because of negatively charged carboxyfluorescein (CF) terminal in the fluorescent PEG lipid. Stepniewski et al. theoretically showed that vesicle membranes containing PEG lipids repel negative ions due to a layer of PEG formed on the membrane, while it attracts positive ions. [36] If the same mechanism can be applied to our case, we think there were two stages in the shape transformation process: (1) Incorporation of PEG lipids into preformed lipid vesicles. At this stage, vesicles incorporate PEG lipids regardless of the functionalized molecules (CF or biotin) and transform into oblate or prolate shapes. (2) Formation of PEG layer: After the PEG lipids were incorporated into vesicles to some extent, they start forming a layer of PEG chains sticking out of the vesicle membrane. At this stage, the PEG layer expels negatively charged fluorescent PEG lipids, while non-charged biotinylated PEG lipids can be still incorporated and vesicles continue to transform to budded/divided shapes.

Due to the relatively low budding/dividing transformation rate of fluorescent PEG lipids, we report below the behavior of vesicle deformation by the biotinylated PEG lipids only.

It is known that shape transformations described above can be induced by osmotic pressure difference across vesicle membranes. [25, 37] However, in this experiment, it is speculated that fusions of PEG lipid micelles into lipid vesicles gave rise to the transformations because of following reasons: (1) osmotic pressure differences across vesicle membranes are too small. The molecules that potentially induce an osmotic pressure difference are TA647 and PEG lipids, which were present in the inside of the vesicle and in the external buffer solution, respectively. The concentrations of both molecules were however quite small (1 *μ*M and up to 165 *μ*M, respectively) and not enough to induce shape transformations. The effect of TA647 can be also excluded from the fact that no shape transformations as shown above were observed when the buffer solution containing no PEG lipid was added to the vesicle suspension. (2) The observed series of vesicle shapes can be explained by increased area difference Δ*a* between a bilayer membrane: it is known that, in general, if Δ*a* is increasing (i.e., more lipids in the outer leaflet than the inner one), a vesicle tends to deform into prolate or form daughter vesicles to relax the bending energy of the bilayer membrane. In contrast, it forms a cavity structure similar to red blood cells if Δ*a* is decreasing. [38] The observed transformations clearly belong to the former type of transformation, which strongly indicates that PEG lipid micelles were integrated into vesicles and increased the area difference. We will further discuss the vesicle shapes and area differences in relation to theoretically predicted vesicle shapes.

### Effect of PEG lipid concentration

**Fig 3:**
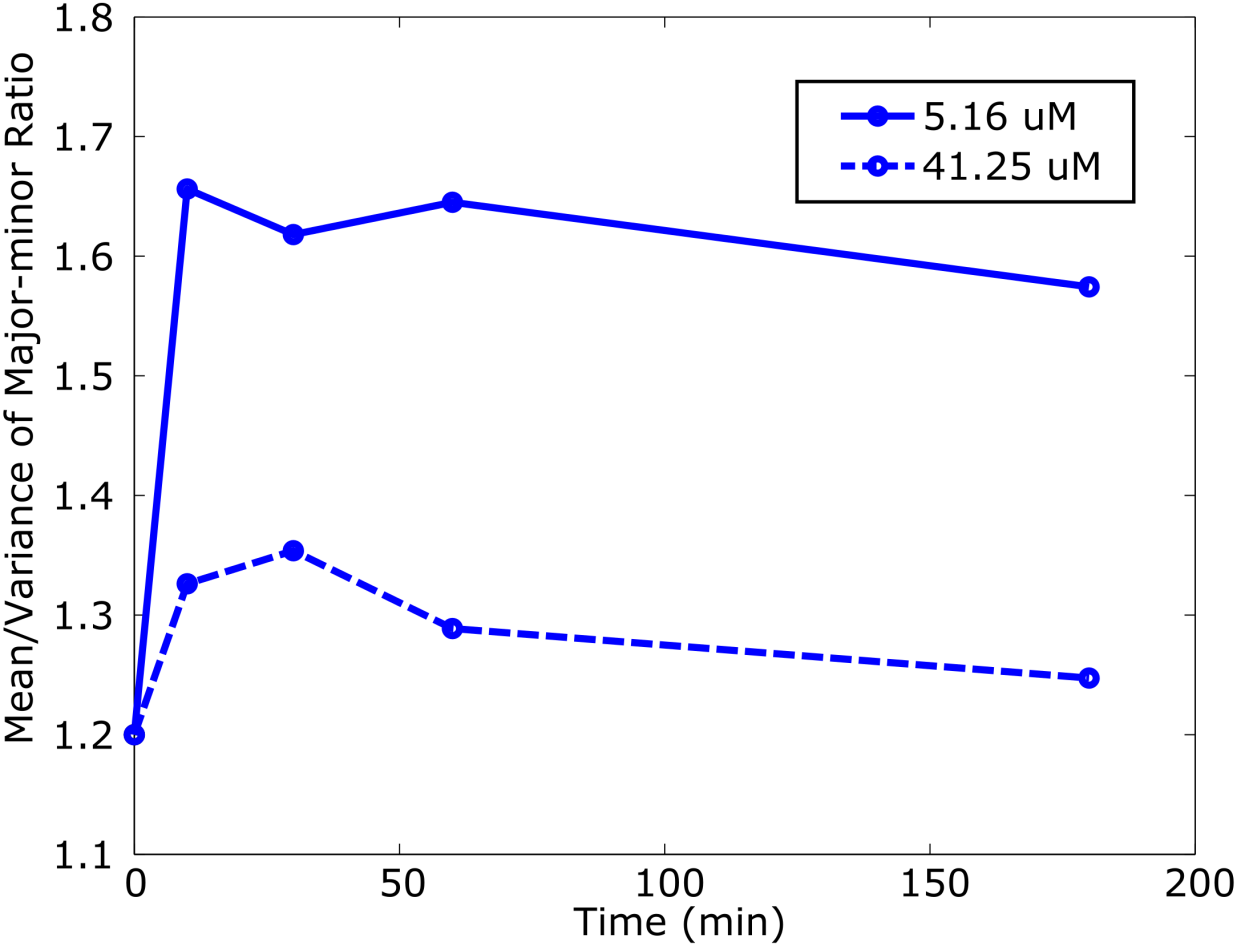
Temporal changes of the mean and variance of the M-m ratio.

Next, we looked into the effect of different PEG lipid concentrations on vesicle shapes. The major-minor ratio (M-m ratio) of deformed vesicles was statistically measured as an index of the outward deformations. For example, if a vesicle is circular (spherical or disc-like), the ratio will be ∼ 1 (the major and minor axes are the same length), whereas it will be larger if the shape is elongated (e.g. cylinder-like). First, we compared the M-m ratio of lipid vesicles with a high and low concentrations of PEG lipid micelles (41.25 and 5.16 *μ*M). Vesicles were exposed to the micelles for different durations (0 min, 10 min, 30 min, 1 h, and 3h). Fig 3 shows the mean of the M-m ratio for this experiment. In both concentrations, the mean M-m ratio immediately increased within 10 min and then gradually decreased as the exposure duration became longer (see S2 Fig for histograms). This indicates that many of the vesicle transformations initiated by PEG lipid micelles completed within the first 10 min, but transformations slowly continued after that. This is in fact consistent with our microscope observation mentioned above.

**Fig 4:**
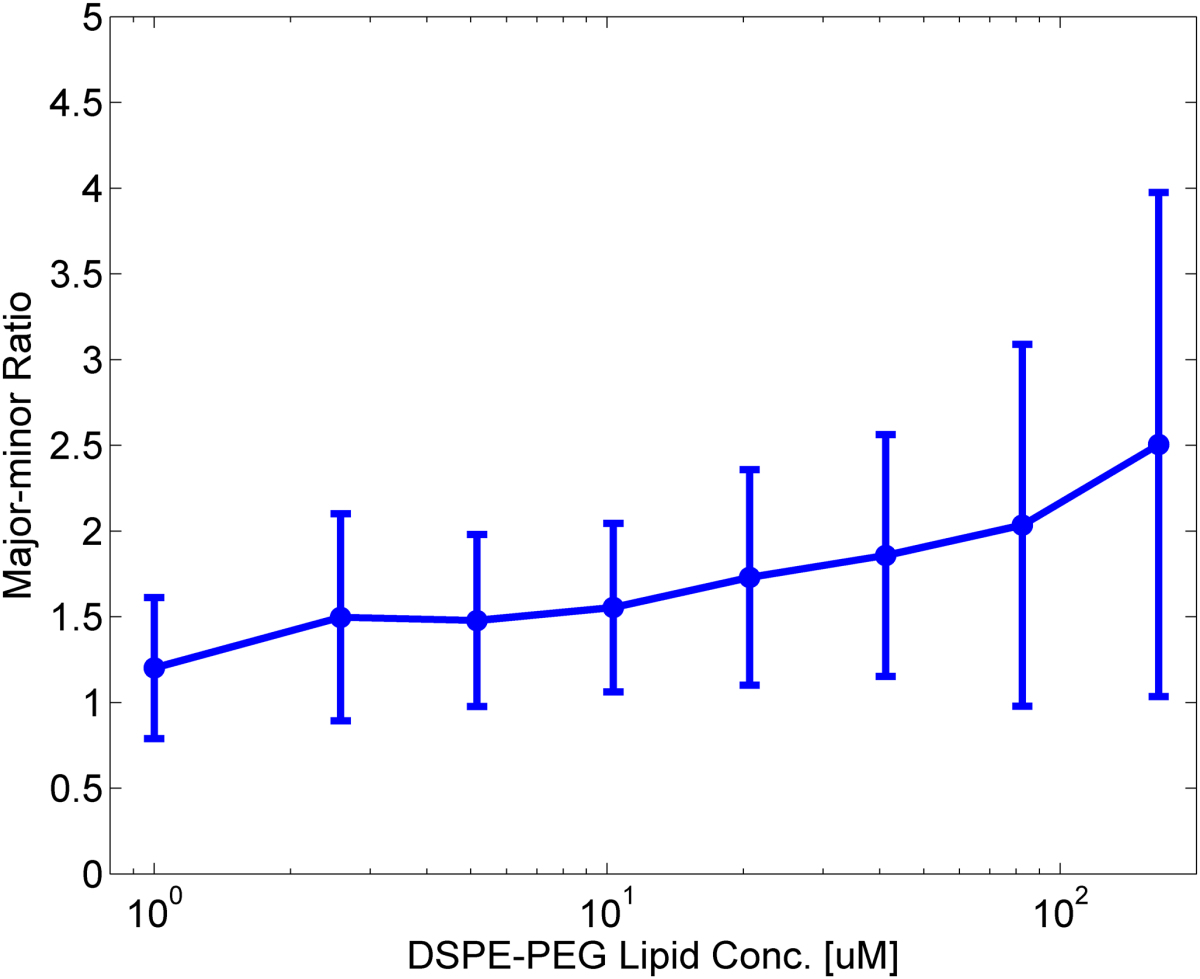
The mean and variance of the M-m ratio against the concentration of PEG lipid micelles.

**Fig 5:**
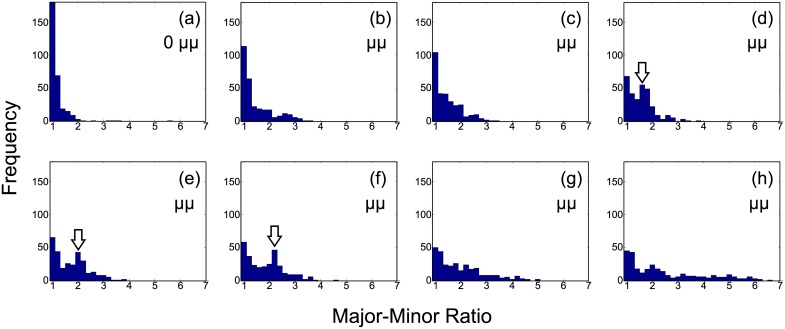
Histograms of the M-m ratio for different concentrations of PEG lipid micelles. (a) 0 *μ*M, (b) 2.58 *μ*M, (c) 5.16 *μ*M, (d) 10.32 *μ*M, (e) 20.63 *μ*M, (f) 41.25 *μ*M, (g) 82.5 *μ*M, and (h) 165 *μ*M. Characteristic peaks were indicated as arrows.

Fig 4 shows the mean the M-m ratio of vesicles exposed to different micelle concentrations. Prior to microscope observation, vesicles suspension was mixed with PEG lipid solution and incubated for 30 min, which would be sufficient for most of shape transformations to complete, as mentioned above. The mean M-m ratio was linearly increasing with the PEG lipid concentration. It is notable that that the error bar (i.e. standard deviation) of the ratio became longer and longer as the concentration of PEG lipids increases. This means that vesicles were deformed into a wide variety of shapes, rather than transformed in to one particular shape. In fact, a wide variety of shapes were observed at a high PEG lipid concentration, while most of the shapes were spherical and round at lower concentrations (See S3 Fig for example microscope snapshot images). These results suggest that more PEG lipids were incorporated into the vesicle membranes at higher concentrations and the area difference increased. In fact, histograms of the M-m ratio gradually changed as the micelle concentration increased, as shown in Fig 5. At the lower PEG lipid concentrations (e.g. Fig 5a and 5b), the histograms show a tall and narrow peak, whereas they are flat and wide at the higher concentrations (e.g. Fig 5g and 5h). This means that the vesicle deformations were dependent on the concentration of micelles: A majority of vesicles were intact (i.e., in spherical shapes and the M-m ratio is ∼ 1) at the lower concentrations. In contrast, the higher the PEG lipid concentration became, the more and more vesicles transformed into non-spherical shapes. This was clearly represented by the disappearing peak at the M-m ratio = 1 as well as the expanding tail in the region of M-m ratio ≫ 1.

### Comparison with theoretical models

In this section we further discuss the vesicle shapes observed and the area difference in relation to theoretical prediction. First, simulated vesicle shapes using the BC model were obtained as follows: an area difference Δ*a* (difference in area between outer and inner membranes) and reduced volume *v* (relative volume compared to a sphere with the same area) were given as constraints and a shape with the minimum bending energy *w*_*b*_ was searched as a parameter optimization process among other possible shapes satisfying Δ*a* and *v* constraints (for details, see [25]). Hence, a vesicle shape with the minimum bending energy *w*_*b*_ was uniquely determined for a given Δ*a* and *v*. A library of vesicle shapes were obtained for constraint parameter pairs of Δ*a* = 0.5–1.6 and v = 0.6–1.0 with 0.01 steps (in total, 4400 conditions).

**Fig 6:**
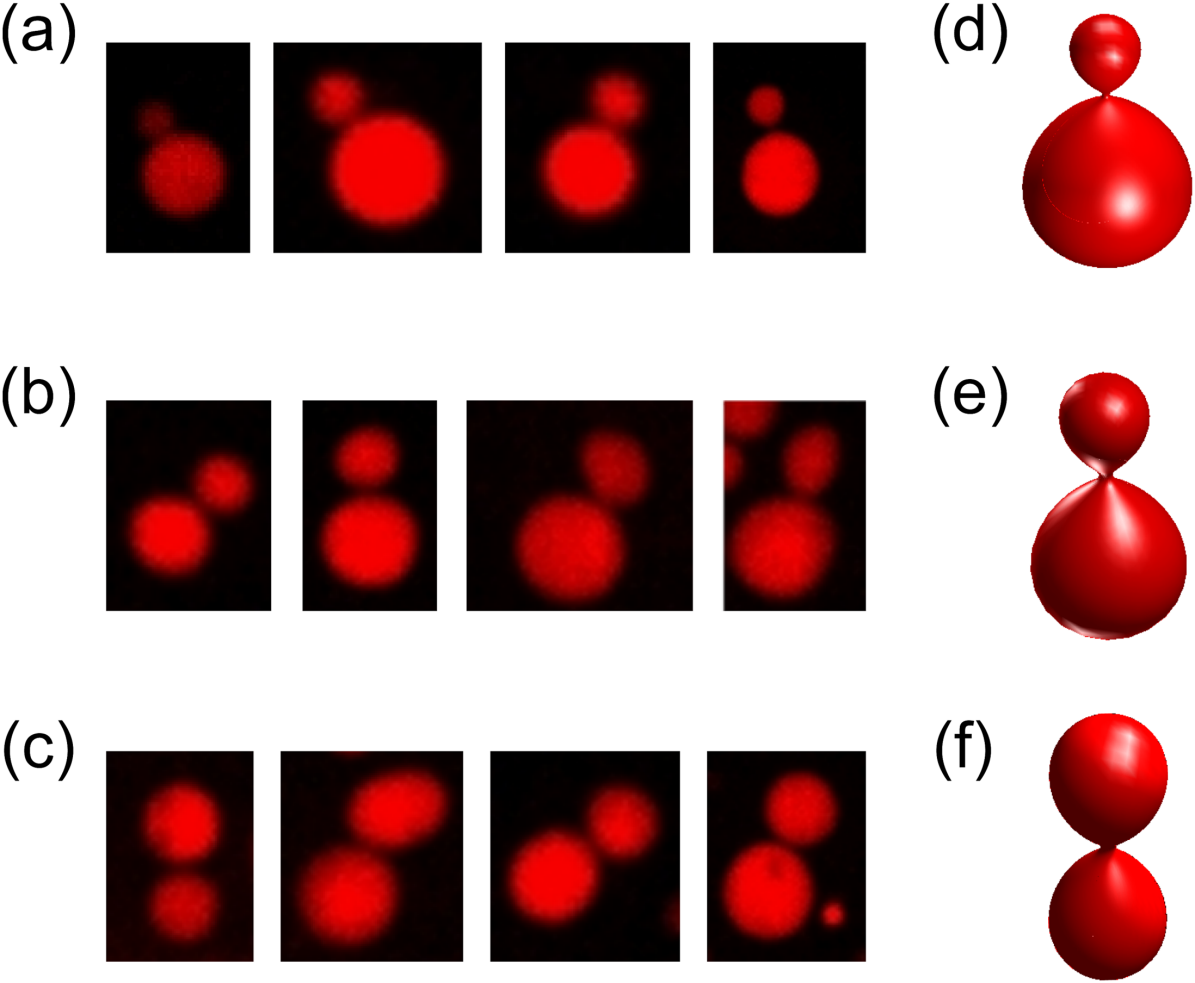
Microscope images of lipid vesicles consisting the peaks (arrowed) of Fig 3. The concentrations of PEG lipid micelles are (a) 10.32*μ*M, (b) 20.63 *μ*M, and (c) 41.25 *μ*M, which correspond to Fig 3f, 3e, and 3d, respectively. Images of simulated vesicles that look similar to observed vesicles are shown on the right. Parameters of the vesicles are: (d) *v* = 0.83, Δ*a* = 1.30, (e) *v* = 0.76, Δ*a* = 1.34, (f) *v* = 0.69, Δ*a* = 1.39.

We then looked into what kind of vesicle shapes consisted of the characteristic peaks observed in Fig 5d-5f (arrowed). A simulated vesicle shape that looked similar to vesicles in a peak was manually picked by comparing microscope images and simulated shapes. Vesicles in the peak region were the ones deformed by PEG lipid micelles because the M-m ratio was at 1.5–2.5. Yet the frequencies were almost equivalent to the ones at the M-m ratio = 1. Fig 6a-6c are examples of raw microscope images of deformed vesicles forming those peaks for each condition. At a lower PEG lipid concentration (Fig 6a), vesicles appeared to have asymmetrically divided into one large and one small vesicles, whereas they were symmetrically divided into two equal-sized vesicles at higher concentration (Fig 6c). Most of vesicles in a peak looked similar in shape. This suggests that the divided vesicle shapes correspond to “local minima” that are energetically favored than other shapes. Käs et al. reported that similar budding transformations occurred when vesicles are gradually heated. Due to different thermal expansivities of inner and outer bilayer membranes, heating causes an increase in Δ*a* (outer membrane expands more than inner one). [5] After Δ*a* is changed, the shape of a vesicle before heating no longer has the minimum bending energy at a new Δ*a*, and hence the vesicle were thermodynamically deformed as an energy relaxation process into another shape to have the minimum energy. The transformations were in fact explained in the BC model. [5] In our case, we can assume vesicles went through a similar process, i.e., an increase of Δ*a*, but by incorporation of PEG lipids into the membrane.

The symmetry of vesicle division can be explained by area difference Δ*a*. Fig 6d-6f show theoretical shapes modeled by the Bilayer-Coupling (BC) model, which look similar to the budded vesicles described above. As seen from the Δ*a* values of the theoretical vesicles, the larger the Δ*a* becomes, the more symmetric the budding of vesicle becomes. Thus, if the observed vesicles are in similar conditions as the corresponding theoretical vesicles, the budded vesicles should have different Δ*a* values depending on the symmetry of the shape. In other words, budded vesicles at the higher concentration condition should have more PEG lipids inserted on the outer leaflet of the membrane. This assumption would be plausible because of following reasons: (1) As the PEG lipid itself is water soluble owing to the large hydrophilic part, PEG lipids were assumed to be spontaneously associating and dissociating to/from lipid vesicle membranes. [27, 28] This means that we can assume there are two possible pathways for PEG lipid incorporation into vesicle membranes.

$$\left[{\text{PEGLipid}}_{\text{micelles}}\right]⇌\left[{\text{PEGLipid}}_{\text{monomer}}\right]⇌\left[{\text{PEGLipid}}_{\text{membrane}}\right]$$ (1)

$$\left[{\text{PEGLipid}}_{\text{micelles}}\right]⇌\left[{\text{PEGLipid}}_{\text{membrane}}\right]$$ (2)

where [PEGLipid_micelles_], [PEGLipid_monomer_] and [PEGLipid_membrane_] indicate the concentrations of PEG lipid micelles and monomers in the buffer solution, and on the vesicle membranes, respectively. Eq (1) represents a process of the desorption of PEG lipid monomer from micelles and incorporation into vesicle membrane, based on a scheme proposed by Kastantin et al. [27] Eq (2) is a process of direct micelle integration into lipid vesicles. When vesicle shape deformations stopped, we can assume the system reached an equilibrium between the concentrations of PEG lipids in the buffer solution and in the vesicle membranes. In Eq (1), the rate-limiting reaction is believed to be the desorption of PEG lipids from micelles or membranes, which means PEG lipids tend to remain in the membrane once incorporated. [39, 40] When [PEGLipid_micelles_] becomes higher, the equilibrium would be shifted to the right hand side in both equations, which leaves more PEG lipids on the vesicles. The PEG lipid does not flip-flop to the inner membrane due to the PEGylated bulky head group (at least within the timescale of our experiments). Hence, it is likely to stay on the outer leaflet of vesicle membranes and keeps Δ*a* unbalanced. On the other hand, if a direct incorporation of PEG lipid micelles in Eq (2) occurs, it would have disturbed the vesicle membrane, which can result in, for example, temporal pore formation or re-organization of inner/outer membrane compositions to correct unbalanced Δ*a*. However, it would have been much less frequent than the monomer incorporation, considering that monomer incorporation into vesicles should thermodynamically require much less energy than micelle fusion into a membrane. Overall, we can suppose that an outer membrane of a vesicle would have had more PEG lipids incorporated than the inner one (i.e., increase in Δ*a*), which have driven the shape transformation into the budded shapes. In fact, when theoretical vesicle shapes in Fig 6d-6f were compared, Δ*a* of those shapes was increasing as the concentration of the PEG lipid became higher. Although this is a qualitative comparison rather than quantitative, the increasing Δ*a* would partly explain the concentration dependence of vesicle deformation mentioned earlier.

**Fig 7:**
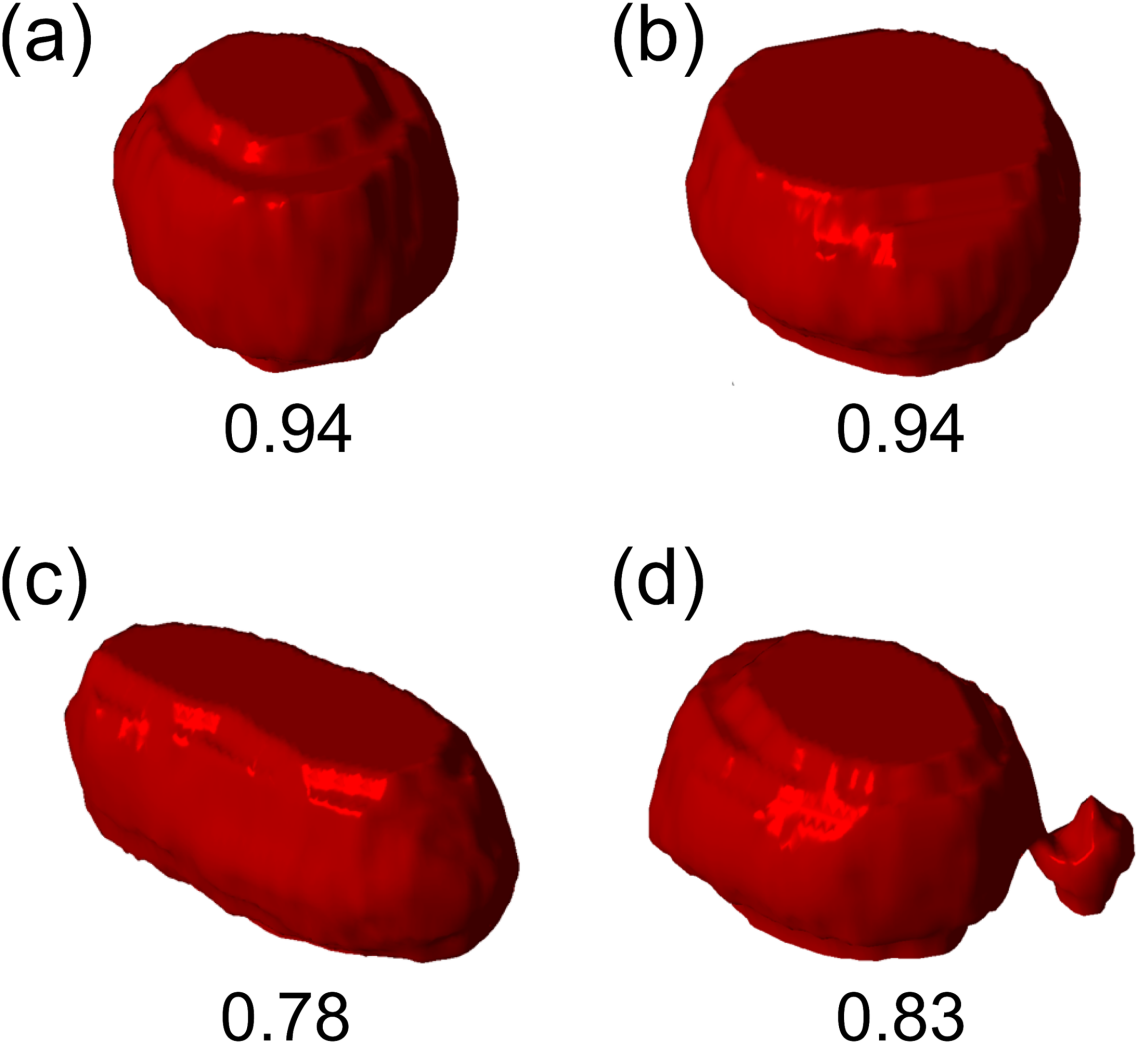
Reconstructed 3D images of a transforming lipid vesicle. (a) immediately after the addition of PEG lipid micelles, (b) after 2 min, (c) after 4 min (d) after 8 min. Numbers below images indicate the reduced volume *v*. The concentration of PEG lipid was 2.58 *μ*M.

Another important point in relation to theoretical vesicles is the reduced volume *v*. As shown in Fig 6, the simulated vesicles all take different *v* as well as Δ*a*. Again, if we assume that the condition of observed vesicles are similar to that of the simulated ones, it suggests that water was lost from the internal of lipid vesicles during shape transformations. To experimentally confirm this, we took stacked images of deforming vesicles and reconstructed three-dimensionally to calculate the reduced volume. Fig 7 shows 3D shapes of a lipid vesicle reconstructed from 2D stacked confocal microscope images. The vesicle, when immediately after PEG lipid micelles were added, was taking a shape slightly deformed from a sphere due to the gravity (Fig 7a). At this point, the reduced volume *v* was close to 1. It then slowly deformed as shown in Fig 1: First, it deformed into an oblate shape (Fig 7b), while maintaining the reduced volume. At 4 min, the vesicle transformed into a prolate and the reduced volume drastically dropped (Fig 7c). The reduced volume gradually recovered as it asymmetrically divided into one large and small vesicles (Fig 7d), which has *v* = 0.83 at 8 min after the addition of micelles.

Taken together with all these findings described above, we speculate that the mechanism of PEG lipid-induced shape transformation can be explained as follows. As we mentioned in a previous section, there would be two stages in a shape transformation from a spherical to budded/divided vesicle: (1) Incorporation of PEG lipids into vesicle membranes and (2) Formation PEG layer on the membrane.

At the first stage, mostly PEG lipid monomers, rather than micelles, may have been inserted into vesicle membranes. This is because of less energy requirement of monomer insertion than micelle integration into vesicle membranes. If a micelle is incorporated into a vesicle membrane, this would be a substantial disruption to the bilayer, which may result in temporal pore formation, as well as loss of water through pores. Re-organization of inner/outer membrane lipid composition may have also occurred due to membrane disruption, which would introduce PEG lipids into inner membrane. However, at the lower concentration of PEG lipids which contains less micelles (Fig 7), the vesicle was deformed but the reduced volume stayed constant (Fig 7a and 7b). This supports our assumption that PEG lipid monomers were mainly inserted while micelles incorporation were rather rare.

Several different processes may have been involved in the second stage. First, PEG lipids continue to be incorporated into the membrane (in the case of biotynylated PEG lipids). At the same time, a layer of PEG would be formed on the vesicle membrane. If PEG lipids are incorporated mostly into the outer membrane, this forms asymmetry across the bilayer. Not only this process increases the area difference Δ*a*, a portion of the PEG polymer would be embedded in the membrane. [36] Stepniewski et al. found in their molecular dynamics simulations that PEG part of a PEGylated lipid can enter a lipid bilayer if the membrane is in the liquid-crystalline phase due to its looser structure compared to the gel phase. In our case, vesicles consists of POPC and POPG, which phase transition temperatures are both -2°C. Thus, PEG polymer may have increased the area difference Δ*a* by being embedded in the outer membrane and driven the shape transformations further. Micelle incorporations and subsequent membrane disruptions may have also occurred at this stage, which caused temporal pores and loss of water from the internal of a vesicle. In fact, noticeable decrease in reduced volume was observed in the later stage of vesicle deformation (Fig 7c and 7d).

## Conclusion

In conclusion, we have shown that shape transformations of lipid vesicles were induced by the bulk-head PEGylated lipid micelles. The types of observed shape transformation were outward deformations, such as cylinder-like prolate shape or division into multiple vesicles. Statistical analyses of microscope images revealed that the degree of deformation was concentration-dependent. Comparisons with theoretically simulated vesicle shapes suggested that one of frequently observed shapes (divided shapes) was energetically-favored than other shapes. These experimental evidences and comparison with theoretical models lead to a potential shape transformation mechanism by PEG lipids, which involves selective incorporation of PEG lipids into vesicle membranes and formation PEG layer on the membrane.

In relation to our motivation for this research (i.e., establishing a method for controlled vesicle shape transformation), it was quite important that the shape transformations induced by PEG lipid micelles were found to be concentration-dependent. This means that vesicles can be induced to deform into a particular shape. Even though deformations of vesicles are in principle stochastic, they would tend to be transformed into energetically-favored shapes as previously reported an also as we showed above. This would potentially provide a way to control shape transformations (particularly, division) of lipid vesicles. It is expected that this method would also enable the investigation into the relation between vesicle transformations and encapsulated molecules. For example, the re-distribution of encapsulated nanomaterials (e.g. *λ*DNA [31]) upon vesicle division can be possible in a more controlled fashion based on the method developed here.

## Supporting Information

S1 Fig A plot of the minimum bending energy *w*_*b*_ against area difference Δ*a* for a fixed reduced volume *v* = 0.80. Four typical shapes are shown in the plot: stomatocyte, oblate, prolate, and bowling pin-like vesicle (left to right), which area differences are *a* = 0.94,1.03,1.12 and 1.25, respectively.
(TIF)

S2 Fig Histograms of the M-m ratio for vesicles exposed to two different concentrations of PEG lipid micelles for various durations. (a) 41.25 *μ*M, 10 min (b) 41.25 *μ*M, 30 min, (c) 41.25 *μ*M, 1 h, (d) 41.25 *μ*M, 3h, (e) 5.16 *μ*M, 10 min, (f) 5.16 *μ*M, 30 min, (g) 5.16 *μ*M, 1 h, and (h) 5.16 *μ*M, 4h.
(EPS)

S3 Fig Snapshot microscope images of lipid vesicles for different concentrations. PEG lipid concentreations are (a) 0 *μ*M, (b) 5.16 *μ*M, (c) 41.25 *μ*M, (d) 165 *μ*M.
(TIF)

S1 Video Time-lapse microscope images of a lipid vesicle. Recording was started immediately after PEG lipid micelles (final concentration 2.58 *μ*M) were added to the solution containing the vesicle. x15 speed.
(WMV)
