# Supplementary figures and images for: Shape Transformations of Lipid Vesicles by Insertion of Bulky-Head Lipids
Source: PLoS One. 2015 Jul 15;10(7):e0132963. doi: 10.1371/journal.pone.0132963 (PMC4503622; doi:10.1371/journal.pone.0132963)

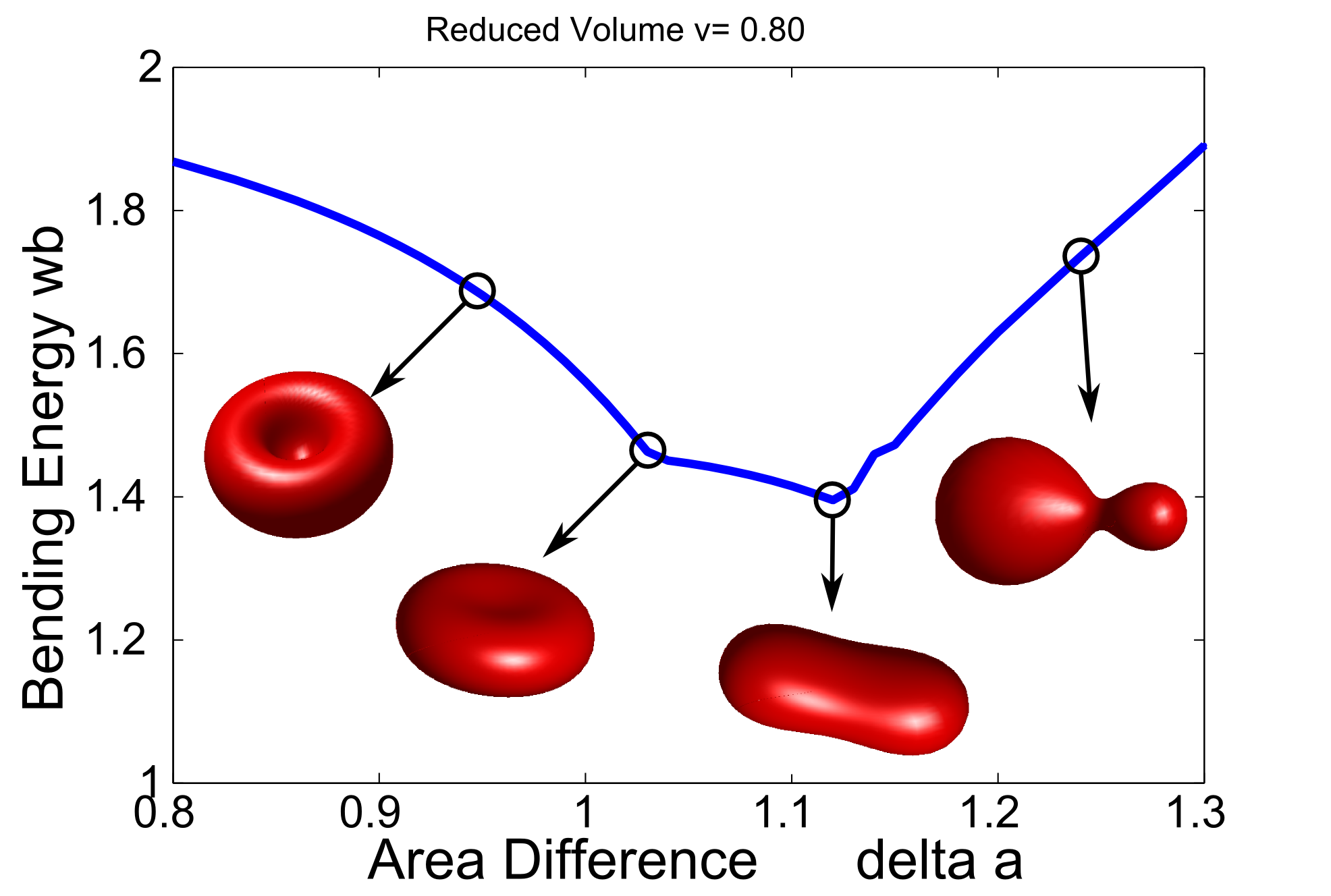

Supplement: S1 Fig — Four typical shapes are shown in the plot: stomatocyte, oblate, prolate, and bowling pin-like vesicle (left to right), which area differences are a = 0.94,1.03,1.12 and 1.25, respectively. (TIF) [file pone.0132963.s001.tif]

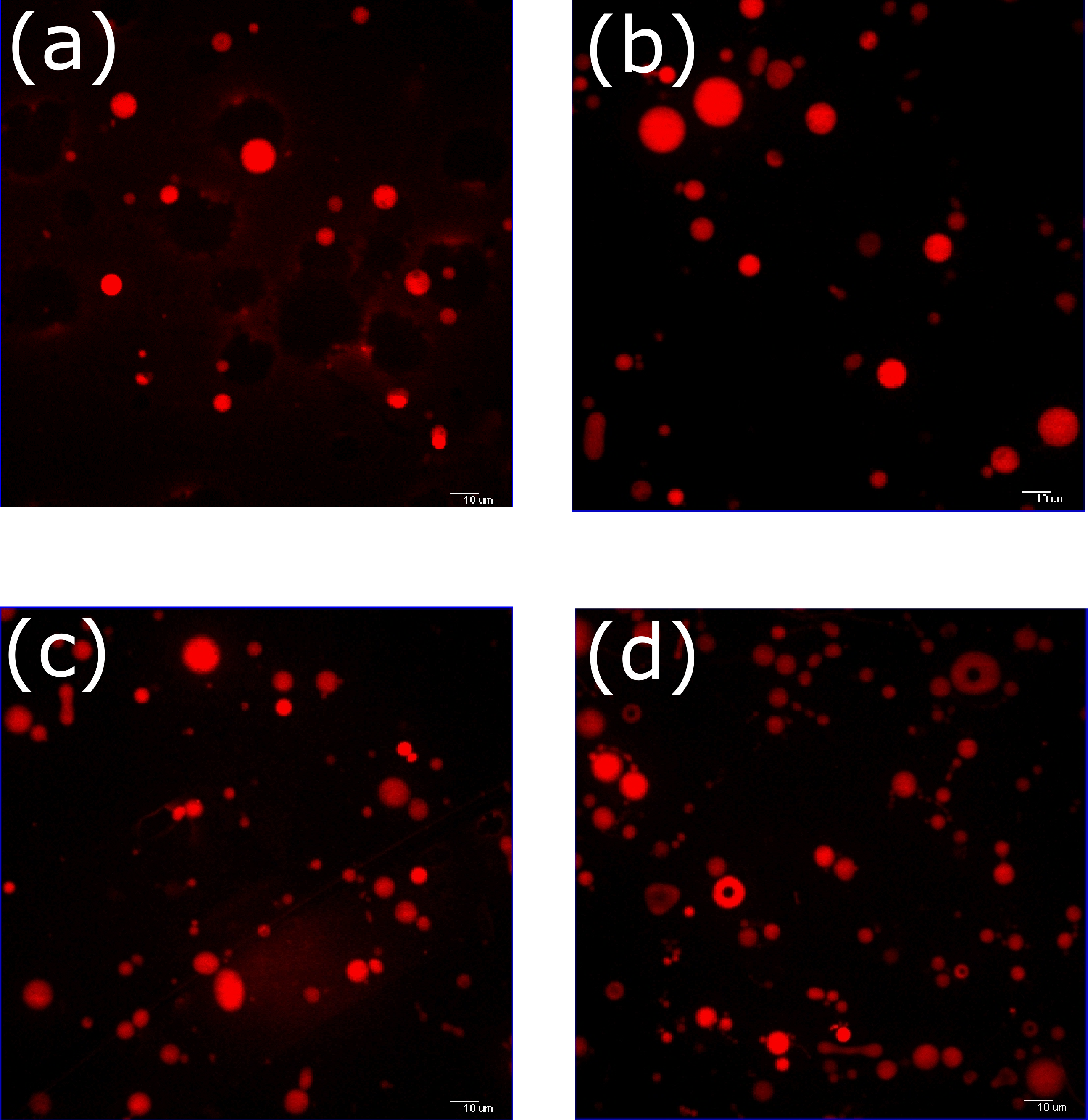

Supplement: S3 Fig — PEG lipid concentreations are (a) 0 μM, (b) 5.16 μM, (c) 41.25 μM, (d) 165 μM. (TIF) [file pone.0132963.s003.tif]
